# Role of renal doppler in children with idiopathic nephrotic syndrome

**DOI:** 10.1186/s12887-025-06492-w

**Published:** 2026-02-03

**Authors:** Doaa El Amrousy, Rana Attalla, Sara Elghoul

**Affiliations:** 1 Pediatrics Department, Faculty of Medicine, Tanta University, Tanta, Egypt; 2 Radiology Department, Faculty of Medicine, Tanta University, Tanta, Egypt

**Keywords:** Idiopathic nephrotic syndrome, Renal resistive index, Pediatric, Steroid resistance, Disease outcome

## Abstract

**Background:**

Renal Doppler can measure intrarenal vascular resistance and may help determine the degree of intrarenal damage, as well as predict subsequent kidney function impairment. However, its utility in children with an underlying kidney disease such as nephrotic syndrome has not been widely examined. This work aimed to measure serial renal resistive index (RI) in pediatric patients with idiopathic nephrotic syndrome to assess its predictive value for steroid resistance and disease outcome.

**Methods:**

This prospective cohort study included 60 patients with idiopathic nephrotic syndrome aged 5 to < 18 years. Renal Doppler was performed on all children, and renal RI was measured at diagnosis, after 1, 3, and 6 months of diagnosis.

**Results:**

The average interlobar renal RI of the right and left kidneys at diagnosis, during follow-up at 1, 3, and 6 months, was significantly higher in steroid-resistant nephrotic syndrome than in the steroid-sensitive nephrotic syndrome. Meanwhile, the estimated glomerular filtration rate (GFR) was significantly lower in steroid-resistant nephrotic syndrome than in steroid-sensitive nephrotic syndrome after 6 months, with no significant difference at diagnosis, after 1 month, or 3 months. The average interlobar RI at diagnosis can predict steroid resistance at a cutoff > 0.60 with 92.31% sensitivity and 85.32% specificity. Moreover, the average interlobar RI after 3 months of follow-up can predict short disease outcome at a cutoff > 0.63 with 84.62% sensitivity and 82.98% specificity.

**Conclusions:**

Renal RI might be an effective non-invasive tool for early prediction and risk stratification of steroid resistance in pediatric patients with idiopathic nephrotic syndrome. Its utility lies in supporting earlier clinical decisions rather than replacing established diagnostic methods.

## Introduction

Nephrotic syndrome is the most common pediatric glomerular disease [1]. It is characterized by heavy proteinuria (proteinuria exceeding 40 mg/m^2^/h or a spot urinary protein-to-creatinine ratio exceeding 2 mg/mg), hypoalbuminemia (albumin level < 2.5 g/dL), edema, and hyperlipidemia, and is classified as idiopathic nephrotic syndrome (INS) when no underlying systemic disease is identified [2]. INS is the predominant form in children, with minimal change disease (MCD) being the most frequent pathological subtype [3].

About 80% − 90% of children with idiopathic nephrotic syndrome show complete remission within the initial 4 weeks of corticosteroid therapy, and are classified as ‘steroid sensitive nephrotic syndrome’ [4, 5]. The remaining are steroid-resistant nephrotic syndrome [6]. It is defined as unresponsiveness to 60 mg/m^2^ body surface area per day for 4 weeks of prednisone therapy [4]. The prognosis of the disease correlates closely with the patients’ response to steroids [7]. Steroid-resistant nephrotic syndrome is complicated by hypertension and impairment of renal function at early stages, and so rapid progression to end-stage kidney disease [8, 9].

Finding a non-invasive tool that can suggest steroid resistance will be of great value in determining disease prognosis. Renal Doppler might be used to achieve this goal by measuring renal resistive index (RI) [10].

Emerging studies suggest it may serve as an early marker of renal functional changes and disease severity, particularly in differentiating steroid-resistant from steroid-sensitive nephrotic syndrome, and potentially identifying risk factors for poor prognosis [11].

Studies have demonstrated that high RI, proteinuria, and hypertension are known risk factors for the progression to chronic kidney disease [11–13]. It may be beneficial from a clinical point of view to have a non-invasive method for predicting steroid resistance and thus support earlier clinical decisions and closer monitoring, rather than waiting four weeks for a kidney biopsy, and adding other aggressive therapies. Therefore, this work aimed to measure serial renal RI in pediatric patients with idiopathic nephrotic syndrome to assess its predictive value for steroid resistance and its utility as a prognostic tool for disease outcome (as defined by KDIGO 2021 criteria). 

## Patients and methods

This prospective cohort study involved 60 pediatric patients with idiopathic nephrotic syndrome aged 5 to < 18 years. The research was conducted after approval by our Ethics Committee (approval code: 36264PR25/1/23). Informed written consent was obtained from the patient’s parents or caregiver. The study was conducted during the period from January 2023 to January 2024.

Exclusion criteria: patients with congenital nephrotic syndrome, secondary causes of nephrotic syndrome, patients with hypotension or hypertension, patients with bradycardia and other arrhythmia, patients with signs of dehydration, patients with fever, high intra-abdominal pressure, and patients on vasoactive drugs (e.g., long-term calcineurin inhibitors), and patients with estimated GFR ˂ 60 ml/min/1.73 m^2^ were excluded from the study depending on single clinical pediatric assesment.

Diagnosis of the initial episode of idiopathic nephrotic syndrome was made based on the presence of heavy proteinuria (spot urinary protein creatinine ratio exceeding 2 mg/mg), hypoalbuminemia (˂2.5 g/dl), edema, and hypercholesterolemia > 200 mg/dl [2].

All children underwent thorough history taking, clinical examination, including blood pressure monitoring, and anthropometric measurements.

Blood and urine samples were taken before starting treatment. Complete blood count (CBC), erythrocyte sedimentation rate (ESR), C-reactive protein (CRP), serum albumin, cholesterol, triglyceride, creatinine, blood urea, and urinary protein creatinine ratio were measured. Kidney biopsy was done for patients with steroid-resistant nephrotic syndrome.

Renal ultrasound and Doppler were done by a single experienced pediatric radiologist (R.A), to minimize inter-observer variability. All children were evaluated in the supine and lateral positions using a Toshiba Aplio ultrasound machine (Toshiba Medical Systems Corporation, Tustine, CA) with a 2–5 MHz probe. An initial B-mode ultrasound was used to assess kidneys’ length, width, cortical thickness, and echogenicity. Renal echogenicity was qualitatively assessed by comparing the cortical echogenicity to the adjacent liver or spleen parenchyma, with Grade 0 being normal and Grades 1–4 representing increased echogenicity (as is standard clinical practice). For Doppler measurement, renal RI was measured as (peak systolic velocity- end diastolic velocity/ peak systolic velocity) at upper, middle, and lower renal interlobar arteries (at medullary pyramids) of both kidneys at diagnosis, after 1, 3, and 6 months. We opted for interlobar arteries over more distal arcuate arteries because of the young age of our patient cohort, where the smaller and more tortuous arcuate arteries are technically challenging to visualize and reliably assess. Doppler angle correction was maintained at less than 60°, and the pulse repetition frequency (PRF) and wall filter settings were optimized to capture low-velocity diastolic flow and avoid aliasing. To increase reproducibility, Doppler spectral patterns of the velocity–time graph were obtained for three times, and an average of these values was taken. All these values were obtained by the ultrasound machine based on the computer algorithm.

All children received standard steroid therapy and were classified into two categories on follow-up, steroid-sensitive nephrotic syndrome and steroid-resistant nephrotic syndrome, based on their clinical response to steroids. The steroid-resistant nephrotic syndrome was defined as persistence of proteinuria despite full-dose steroids daily for 4 weeks [4]. Systolic and diastolic blood pressure percentile, protein creatinine ratio, and estimation of glomerular filtration rate (GFR) by the modified Schwartz formula were done at 1, 3, and 6 months to follow up on the disease progression [14].

### Study outcomes

The primary outcome of the study was the prediction of steroid resistance (SRNS) based on the average renal RI at diagnosis. The secondary outcome was the prediction of short-term disease progression/outcome at 6 months, defined according to the Kidney Disease: Improving Global Outcomes (KDIGO) 2021 Clinical Practice Guideline.

Disease outcome was considered to have progressed upon the first of the following events: a sustained decrease in estimated glomerular filtration rate (eGFR) of 30% or more from the baseline value; the development of end-stage kidney disease (ESKD), requiring chronic dialysis or kidney transplantation; or a sustained increase in proteinuria, as measured by a urine protein-to-creatinine ratio (UPCR) or 24-hour urine protein, that exceeded the predefined remission criteria [2].

The sample size calculation was done by G*Power 3.1.9.2 (Universität Kiel, Germany). According to a previous study [11], the mean ± SD of RI of the middle pole of the right kidney was 0.56 ± 0.07 in steroid-sensitive nephrotic syndrome and 0.60 ± 0.08 in steroid-resistant nephrotic syndrome. The sample size was based on the following considerations: 1.015 effect size, 95% confidence limit, 95% power of the study, and three cases were added to overcome the dropout. Therefore, we recruited 60 patients in our study.

### Statistical analysis

Statistical analysis was done by SPSS v27 (IBM©, Armonk, NY, USA). The Shapiro-Wilks test and histograms were used to evaluate the normality of the distribution of data. Quantitative parametric data were presented as mean and standard deviation (SD) and were analyzed by unpaired student t-test. Qualitative variables were presented as frequency and percentage (%) and analyzed using the Chi-square test or Fisher’s exact test when appropriate. Comparing means of the same group at different follow-up time points was performed using repeated one-way analysis of variance (ANOVA), and significance difference between the means in each time point was performed using a post hoc test. The receiving operating characteristics (ROC) curve was drawn to assess the predictive value of RI to predict steroid responsiveness and short-term disease outcome. A multivariate logistic regression analysis was performed to identify predictors of steroid resistance. A two-tailed *P* value < 0.05 was considered statistically significant.

## Results

Steroid-sensitive nephrotic syndrome patients constitute 71.7% (*n* = 43) and steroid-resistant nephrotic syndrome 28.3% (*n* = 17) of the total patients. Age, sex, weight Z score, height Z score, and body mass index (BMI) Z score were insignificantly different between the groups. Regarding renal biopsy results in steroid-resistant nephrotic syndrome, focal segmental glomerulosclerosis was the most common pathology in 70.59%. (Table 1).

**Table 1:** Demographic data and renal biopsy of the studied groups

Parameters		SSNS group (*n* = 43)	SRNS group (*n* = 17)	*P*-value
Age (years)		9.49 ± 3.77	11.62 ± 3.2	0.069
Sex	Male	27 (62.79%)	11 (64.71%)	0.890
	Female	16 (37.21%)	6 (35.29%)	
Weight Z score		0.55 ± 1.86	0.67 ± 1.51	0.828
Height Z score		1.09 ± 2.27	1.38 ± 3.32	0.723
BMI Z score		1.24 ± 0.98	1.86 ± 1.18	0.058
Renal biopsy	Not performed	---	0 (0%)	**---**
	MCNS	---	4 (23.53%)	
	FSGS	---	12 (70.59%)	
	Diffuse mesangial proliferative GN	---	1 (5.88%)	

*SSNS* steroid-sensitive nephrotic syndrome, *SRNS* steroid-resistant nephrotic syndrome, *BMI* body mass index, *MCNS* Minimal change nephrotic syndrome, *FSGS* Focal segmental glomerulosclerosis, *GN* glomerulonephritis

Regarding the clinical and laboratory investigations done before therapy, diastolic blood pressure, systolic blood pressure, estimated GFR, platelet count, haemoglobin (Hb), total leucocytic count, ESR, CRP, creatinine, urea, albumin, cholesterol, and triglycerides were comparable in both groups. (Table 2).

**Table 2:** Clinical and laboratory investigations of the studied groups at diagnosis

Parameters	SSNS group (*n* = 43)	SRNS group (*n* = 17)	*P*-value
SBP (percentile)	60.36 ± 24.76	57.23 ± 24.99	0.689
DBP (percentile)	54.15 ± 19.49	60.77 ± 25.07	0.313
HR (b/m)	85.2 ± 15.3	83.4 ± 17.6	0.561
Estimated GFR	113.38 ± 8.93	109.62 ± 7.05	0.166
Platelet count (x10^3^/mm^3^)	407.26 ± 135.03	394.23 ± 144.65	0.763
Hb (g/dL)	11.6 ± 1.22	11.32 ± 1.39	0.486
TLC (cell/mm^3^)	9641.72 ± 6044.21	10,550 ± 5630.82	0.629
ESR 1st hour (mm)	73.28 ± 15.58	77.38 ± 17.63	0.417
CRP (mg/l)	8.83 ± 0.96	9.38 ± 0.87	0.066
Creatinine (mg/dL)	0.93 ± 0.43	1.12 ± 0.49	0.182
Urea (mg/dL)	40.4 ± 13.19	48.38 ± 16.74	0.074
Albumin (g/dL)	1.59 ± 0.34	1.42 ± 0.26	0.102
Cholesterol (mg/dL)	445.62 ± 130.3	520.77 ± 165.15	0.088
Triglycerides (mg/dL)	269.32 ± 133.49	320.77 ± 150.64	0.236

*SSNS* steroid sensitive nephrotic syndrome, *SRNS* steroid resistant nephrotic syndrome,* SBP* systolic blood pressure, *DBP* diastolic blood pressure, *HR* heart rate, *GFR* glomerular filtration rate, *Hb* Hemoglobin, *TLC* Total leukocyte count, *ESR* Erythrocyte sedimentation rate, *CRP* C-reactive protein

Concerning the sonographic findings, length, breadth, cortical thickness, and echogenicity were insignificantly different between the groups in both the right and left kidney. (Table 3).

**Table 3:** Length, breadth, cortical thickness, and echogenicity of the studied groups

Parameters		Right kidney			Left kidney		
		SSNS	SRNS	*P*-value	SSNS	SRNS	*P*-value
Length (cm)		9.17 ± 1.26	8.54 ± 1.56	0.134	8.94 ± 1.44	8.69 ± 1.44	0.590
Breadth (cm)		3.83 ± 0.54	3.55 ± 0.53	0.113	4.57 ± 0.75	4.16 ± 0.87	0.096
Cortical thickness (cm)		0.98 ± 0.43	0.91 ± 0.4	0.608	1.02 ± 0.33	1.11 ± 0.51	0.440
Echogenicity	Normal	24 (55.81%)	5 (29.41%)	0.064	32 (74.42%)	9 (52.94%)	0.076
	1	12 (27.91%)	8 (47.06%)		9 (20.93%)	5 (29.41%)	
	2	7 (16.28%)	4 (23.53%)		2 (4.65%)	3 (17.65%)	
	3	0 (0%)	0 (0%)		0 (0%)	0 (0%)	
	4	0 (0%)	0 (0%)		0 (0%)	0 (0%)	

*SSNS* steroid-sensitive nephrotic syndrome, *SRNS* steroid-resistant nephrotic syndrome

As regards the renal RI values at diagnosis, the average interlobar RI of the right and left kidneys was significantly higher in the steroid-resistant nephrotic syndrome group than in the steroid-sensitive nephrotic syndrome group. (Table 4).

**Table 4:** Resistive index of the studied groups at diagnosis

Interlobar RI	Right kidney			Left kidney		
	SSNS (*n* = 43)	SRNS (*n* = 17)	*P*-value	SSNS (*n* = 43)	SRNS (*n* = 17)	*P*-value
Upper	0.59 ± 0.01	0.61 ± 0.04	0.059	0.58 ± 0.02	0.60 ± 0.03	0.061
Middle	0.61 ± 0.03	0.63 ± 0.03	0.057	0.59 ± 0.01	0.61 ± 0.03	0.054
Lower	0.60 ± 0.02	0.61 ± 0.03	0.095	0.60 ± 0.02	0.61 ± 0.03	0.161
Average	0.60 ± 0.01	0.62 ± 0.02	0.008	0.59 ± 0.01	0.62 ± 0.03	0.001

*SSNS* steroid sensitive nephrotic syndrome, *SRNS* steroid resistant nephrotic syndrome, *RI* resistive index

**Fig. 1:**
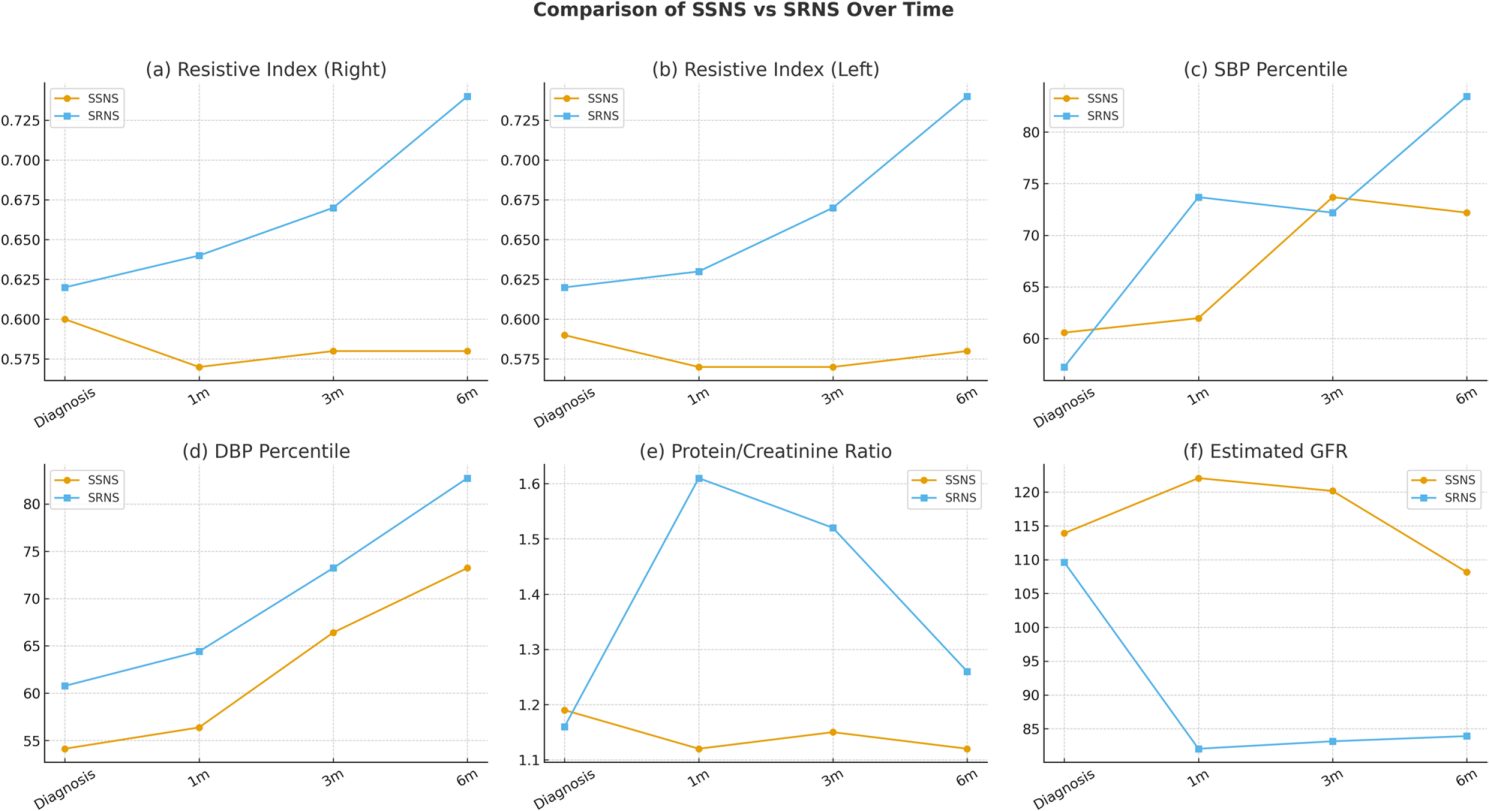
Comparison of patients with steroid-sensitive nephrotic syndrome and those with steroid-resistant nephrotic syndrome across different parameters over time. (**a**) resistive index in the right kidney, (**b**) resistive index in the left kidney, (**c**) systolic blood pressure, (**d**) Diastolic blood pressure, (**e**) Protein/creatinine ratio, and (**f**) estimated GFR

The average RI of the right and left kidneys was significantly higher at diagnosis, 1, 3, and 6 months in steroid-resistant nephrotic syndrome than in steroid-sensitive nephrotic syndrome *(P* < 0.05). Meanwhile, the other parameters, including systolic and diastolic blood pressure percentiles, and estimated GFR were insignificantly different between both groups at diagnosis, 1, and 3 months, but became significantly different after 6 months of follow-up (higher systolic and diastolic blood pressure percentiles in steroid-resistant nephrotic syndrome than steroid-sensitive nephrotic syndrome and lower estimated GFR values in steroid-resistant nephrotic syndrome than steroid-sensitive nephrotic syndrome). Protein/Creatinine ratio was significantly higher at 1, 3, and 6 months in the steroid-resistant nephrotic syndrome group than in the steroid-sensitive nephrotic syndrome group (*P* < 0.001). (Fig. 1).

**Fig. 2:**
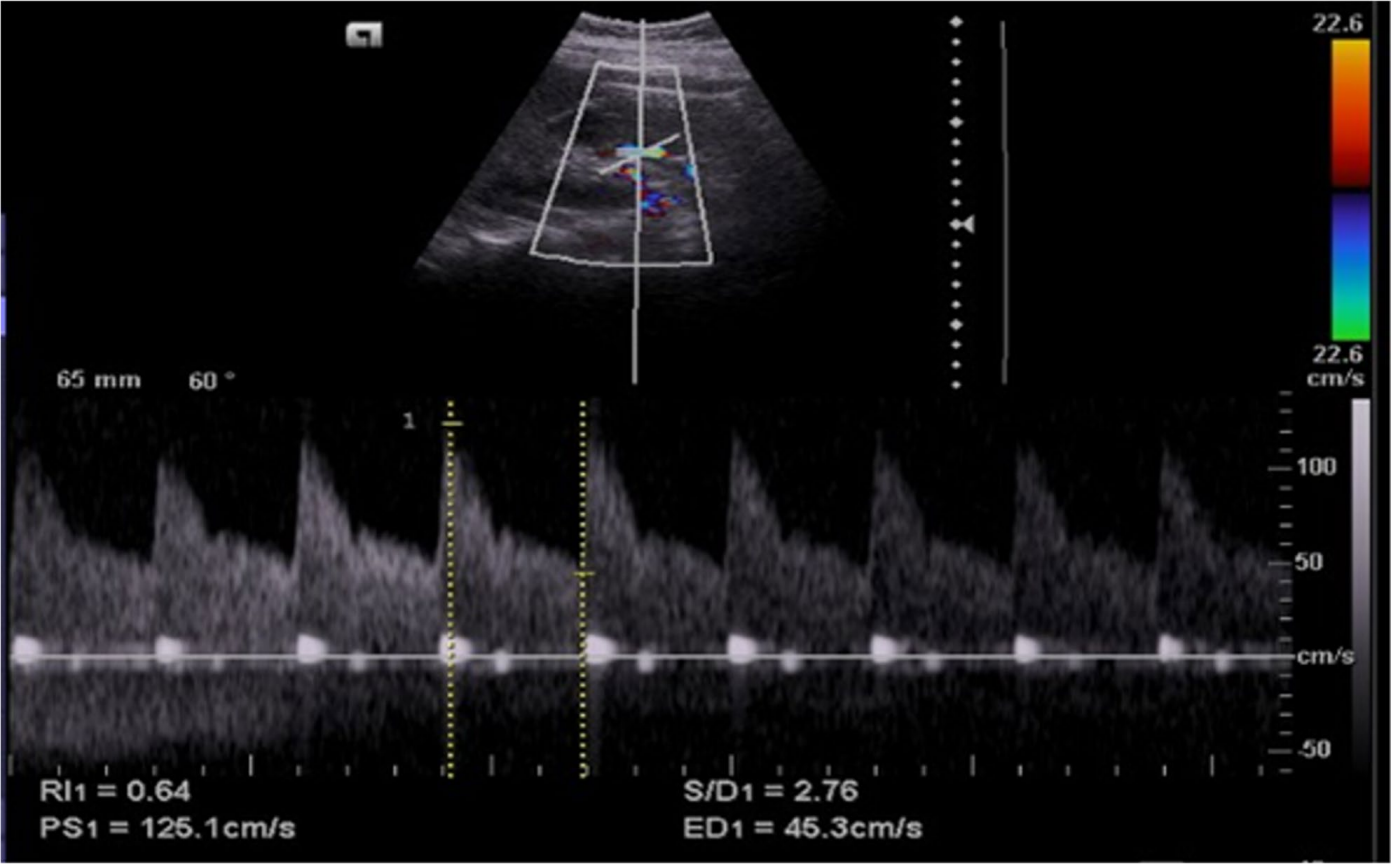
Renal Doppler ultrasound of the right kidney middle pole (at the interlobar artery) in a steroid-sensitive nephrotic syndrome patient (RI = 0.64)

**Fig. 3:**
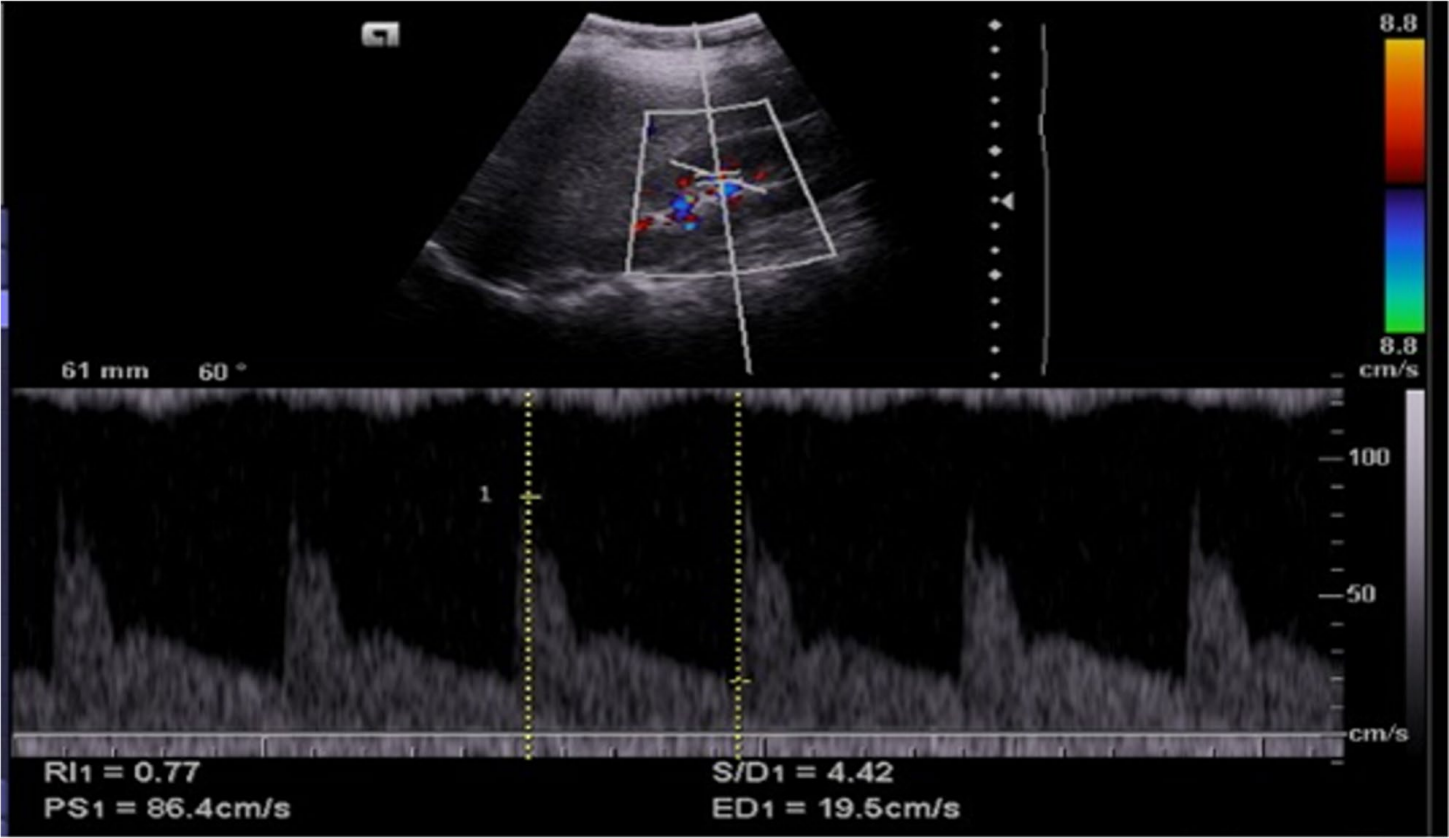
Renal Doppler ultrasound of the right kidney middle pole (at the interlobar artery) in a steroid-resistant nephrotic syndrome patient (RI = 0.77)

Figure 2 showed renal Doppler ultrasound of the right kidney middle pole (at interlobar artery) in the steroid-sensitive nephrotic syndrome patient (RI = 0.64), while (Fig. 3) showed renal Doppler ultrasound of the right kidney middle pole (at interlobar artery) in the steroid-resistant nephrotic syndrome patients (RI = 0.77).

**Fig. 4:**
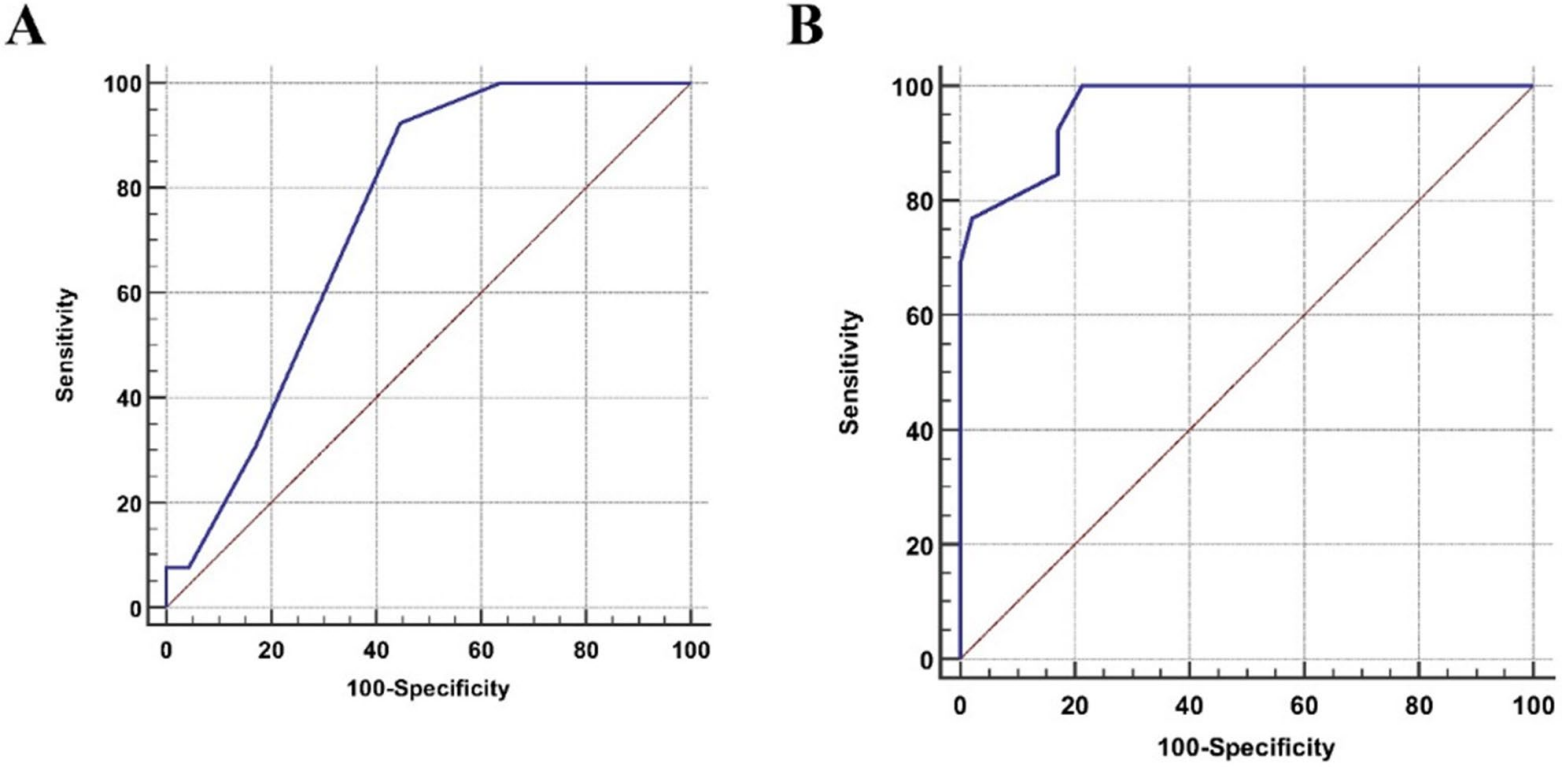
**A** ROC curve of the average interlobar RI at diagnosis to predict steroid resistance as (AUC = 0.844, *P* value < 0.001) at a cutoff > 0.6 with 92.31% sensitivity and 85.32% specificity. **B** ROC curve of the average interlobar RI after 3 months of follow-up to predict progression of the disease (AUC = 0.964, *P* value < 0.001) at a cutoff > 0.63 with 84.62% sensitivity and 82.98% specificity

Additionally, the average interlobar RI at diagnosis can predict steroid resistance (area under the curve [AUC] = 0.844, with 95% CI (0.686–0.899), *P* value < 0.001) at a cutoff > 0.6 with 92.31% sensitivity (95% CI (64-99.8)) and 85.32% specificity (95% CI (74.3–95.2)). (Fig. 4A).

Also, average interlobar RI after 3 months of follow-up can early predict the disease outcome (AUC = 0.964, 95% CI (0.881–0.995), *P* value < 0.001) at a cutoff > 0.63 with 84.62% sensitivity (95% CI (54.6–98.1) ) and 82.98% specificity (95% CI (69.2–92.4)). (Fig. 4B).

Regression analysis revealed that systolic and diastolic blood pressure percentiles, proteinuria, and renal RI at diagnosis were independent predictors of steroid resistance. While age was not. (Table 5).

**Table 5:** Logistic regression for predictors of steroid resistance

Variables	Odds ratio	95% CI	*P*-value
Age	1.164	0.98–1.38	0.075
Basel SBP	1.0353	1.0008–1.0711	0.045
Basel DBP	1.0442	1.0009–1.0894	0.046
Protein/ creatinine ratio	2.3923	1.0965–5.2195	0.028
Renal RI at diagnosis	3.890	1.234–12.296	0.001

*CI* Confidence interval, *SBP* systolic blood pressure, *DBP* diastolic blood pressure, *RI* resistive index

## Discussion

Steroid-resistant nephrotic syndrome is of great clinical importance as it progresses rapidly to end-stage kidney disease [15]. Predicting steroid responsiveness and future decline in kidney function is important for subsequent therapeutic decisions. Renal Doppler can measure intrarenal vascular resistance and may help determine the degree of intrarenal damage and predict subsequent kidney function impairment [10]. Its predictive value has been supported by recent studies, such as that by Rajangam et al. [16], who found that renal RI is a useful early marker in pediatric conditions, including sepsis-associated acute kidney injury. However, its utility in children with an underlying kidney disease such as nephrotic syndrome has not been widely examined. Our study contributes to this limited body of literature by providing longitudinal serial measurements of the Renal RI and predictive cutoff point of its value.

Our study included 60 children diagnosed with idiopathic nephrotic syndrome; steroid-sensitive nephrotic syndrome patients constituted 71.7% and steroid-resistant nephrotic syndrome constituted 28.3%. This was consistent with the rates reported in similar cohorts [17–19], suggesting our findings are representative of the disease’s general epidemiology. The demographic features of our patients, including mean age and male predominance, also aligned with previous studies [11, 20], although our male-to-female ratio was less pronounced than that reported by Swarnim et al. [11], which may be attributed to differences in the age groups studied. Notably, in contrast to Swarnim et al. [11], who found minimal change nephrotic syndrome to be the main histopathological finding in their patients with steroid-resistant nephrotic syndrome, our most common histological finding in these patients was focal segmental glomerulosclerosis (70.59%). This difference underscores the impact of regional, genetic, and ethnic variability in disease characteristics and highlights the importance of localized studies to understand the global epidemiology of SRNS.

Regarding radiological examination by ultrasound, this study showed that the kidneys were more echogenic in steroid-resistant nephrotic syndrome than in steroid-sensitive nephrotic syndrome; however, this difference did not reach a significant level. This was in agreement with the results of other researchers [11, 19]. This might be a feature of steroid-resistant nephrotic syndrome, which needs to be repeated on follow-up to discover if it is a transient feature or a sign of disease progression to chronic kidney disease. Furthermore, Our study demonstrated that the mean renal RI values in patients with steroid-resistant nephrotic syndrome were significantly higher than those in steroid-sensitive nephrotic syndrome in both left and right kidneys, a finding that is both biologically plausible and consistent with recent research [21, 22], which shows that RI reflects not only intrarenal resistance but also systemic cardiovascular changes like pulse pressure and vascular compliance. While the initial differences in mean RI values at diagnosis were statistically significant, we believe that the more pronounced and clinically meaningful differences observed at the 3 and 6-month time points are more indicative of the progressive intrarenal vascular resistance and subsequent fibrosis that distinguish steroid-resistant nephrotic syndrome. These later findings provide stronger biological plausibility and highlight that the true prognostic value of RI in SRNS lies not in a single baseline measurement but in its changes and increases over time.

While other studies have also found a significant association between elevated renal RI and steroid-resistant nephrotic syndrome [11, 20], our methodology offers a unique contribution. Our use of averaged renal RI values from three different interlobar artery sites in both kidneys provides a more comprehensive and likely more accurate representation of the overall intrarenal resistance, as supported by Kajal et al. [23], who emphasize the importance of consistent technical parameters. We chose to measure renal RI at the interlobar arteries rather than the more distal arcuate arteries because these more proximal vessels are technically easier to assess in young patients, ensuring greater measurement accuracy and reproducibility, as noted in recent literature on improved ultrasound techniques by Jeon et al. [24]. A key strength of our study is the longitudinal follow-up, which allowed us to observe changes in renal RI over time. We demonstrated that the average renal RI in SRNS patients increased progressively over the first three months, while systemic markers such as estimated GFR, systolic blood pressure, and diastolic blood pressure percentiles did not show significant differences between the groups until the six-month follow-up. This finding is highly significant as it suggests that renal RI is an early predictor of subclinical changes, capable of detecting hemodynamic abnormalities before they manifest as measurable changes in kidney function or blood pressure.

In our multivariate regression analysis, we found that systolic and diastolic blood pressure, protein/creatinine ratio, and renal RI were independent predictors of steroid-resistance *(P* < 0.05). This key finding highlights that both systemic factors, such as blood pressure, and direct markers of kidney damage, such as proteinuria, are uniquely linked to intrarenal vascular resistance. This suggests that the predictive power of renal RI is further enhanced when combined with these readily available systemic markers, offering a more comprehensive tool for predicting outcome.

Interestingly, the average interlobar RI at diagnosis can predict steroid resistance at a cutoff > 0.6 with 92.31% sensitivity and 85.32% specificity. Moreover, the average interlobar RI after 3 months of follow-up can early predict the short-term outcome of the disease at a cutoff > 0.63 with 84.62% sensitivity and 82.98% specificity. Our finding of a lower optimal cutoff (> 0.60) in our 5 to ˂18 year-old cohort suggests that the predictive threshold may be age-specific. The threshold of > 0.60 was determined by ROC analysis for this specific disease cohort to serve as an age-specific, disease-specific relative discriminator, acknowledging the physiological overlap of RI values in children but providing a validated cutoff for risk stratification in this pediatric age range. This highlights the value of our study in providing a validated cutoff for a specific pediatric age range, thereby informing future clinical practice.

In summary, our study confirms the value of renal RI as a non-invasive tool for assessing intrarenal vascular resistance in children with nephrotic syndrome. By using a comprehensive measurement protocol and providing longitudinal serial measurements of renal RI, we have demonstrated that renal RI can serve as an early, clinically valuable predictor of steroid resistance and short disease outcome. While RI cannot replace histology or genetic testing, it serves as a valuable adjunct tool for risk stratification and to predict SRNS at diagnosis that may support decisions for earlier biopsy, closer monitoring, or consideration of alternative immunosuppression.

The limitations of our study include the relatively small sample size, a single-center design, and the short duration of follow-up. We acknowledge that RI’s reliability is dependent on the technique and operator experience, and its values overlap between SSNS and SRNS. Furthermore, future larger, multi-center studies with different age and ethnic groups are recommended to validate our results.

## Conclusions

Renal RI might be an effective non-invasive tool in the prediction of steroid resistance and short-term outcome of the disease in pediatric patients with idiopathic nephrotic syndrome.

## Data Availability

All data analyzed during the current study are available from the corresponding author on reasonable request.
